# Cardio-oncology: an overview on outpatient management and future developments

**DOI:** 10.1007/s12471-018-1148-7

**Published:** 2018-08-23

**Authors:** A. J. Teske, M. Linschoten, J. A. M. Kamphuis, W. R. Naaktgeboren, T. Leiner, E. van der Wall, J. Kuball, A. van Rhenen, P. A. Doevendans, M. J. Cramer, F. W. Asselbergs

**Affiliations:** 1 Department of Cardiology, Division of Heart and Lungs, University Medical Centre Utrecht, Utrecht, The Netherlands; 2 Department of Radiology, Utrecht University Medical Centre, Utrecht, The Netherlands; 3 Department of Medical Oncology, University Medical Centre Utrecht, Utrecht, The Netherlands; 4 Laboratory of Translational Immunology, University Medical Centre Utrecht, Utrecht, The Netherlands; 5 Department of Haematology, University Medical Centre Utrecht, Utrecht, The Netherlands; 6 Netherlands Heart Institute, Utrecht, The Netherlands; 7 Durrer Centre for Cardiovascular Research, Netherlands Heart Institute, Utrecht, The Netherlands; 8 Institute of Cardiovascular Science, Faculty of Population Health Sciences, University College London, London, UK; 9 Farr Institute of Health Informatics Research and Institute of Health Informatics, University College London, London, UK

**Keywords:** Cardiotoxicity, Chemotherapy, Cardio-oncology, Heart failure

## Abstract

Recent advances in the early detection and treatment of cancer have led to increasing numbers of cancer survivors worldwide. Nonetheless, despite major improvements in the outcome of these patients, long-term side effects of radio- and chemotherapy affect both patient survival and quality of life, independent of the oncological prognosis. Chemotherapy-related cardiac dysfunction is one of the most notorious short-term side effects of anticancer treatment, occurring in ~10% of patients. Progression to overt heart failure carries a strikingly poor prognosis with a 2-year mortality rate of 60%. Early detection of left ventricular damage by periodic monitoring and prompt initiation of heart failure treatment is key in improving cardiovascular prognosis. To meet the growing demand for a specialised interdisciplinary approach for the prevention and management of cardiovascular complications induced by cancer treatment, a new discipline termed cardio-oncology has evolved. However, an uniform, multidisciplinary approach is currently lacking in the Netherlands. This overview provides an introduction and comprehensive summary of this emerging discipline and offers a practical strategy for the outpatient management of this specific patient population.

**Electronic supplementary material:**

The online version of this article (10.1007/s12471-018-1148-7) contains supplementary material, which is available to authorized users.

## Introduction

Advances in the early detection and treatment of cancer have led to increasing numbers of cancer survivors worldwide [1, 2]. Nonetheless, despite this substantial progress, long-term side effects of anticancer treatment can affect patient survival and quality of life considerably. Chemotherapy-related cardiac dysfunction (CTRCD) is one of the most notorious short-term side effects of anticancer treatment, occurring in ~10% of patients [3]. To meet the growing demand for a specialised interdisciplinary approach for the prevention and management of cardiovascular complications, a new discipline termed cardio-oncology has emerged since the late 1990s [4]. Cardiovascular toxicity due to chemo- and radiotherapy manifests itself in many other forms beyond myocardial dysfunction including, for example, hypertension, arrhythmias and valvular and coronary artery disease; these forms of toxicity fall outside the scope of this review [5]. However, the main focus of this overview is on the direct cardiotoxic effects of chemotherapy on cardiomyocyte survival. In this article, we aim to provide the clinical cardiologist, haematologists, and oncologists with an overview of this emerging discipline and share our current knowledge regarding the practical implementation of risk stratification, screening, and treatment of CTRCD. Suggested further reading on the following topics, as well as those that fall outside the scope of this overview, are summarised in Tab. 1.

**Table 1:** Suggested further reading

Ref. no	Author	Year	Topic	Description
[6]	Rochette	2015	Pathophysiology	Cardiotoxic mechanisms of anthracyclines and trastuzumab
[7]	Lenneman	2016	Pathophysiology	Overview of most common anticancer treatments and their mechanism of cardiotoxicity
[8]	Moslehi	2016	Targeted cancer therapy	Overview of cardiovascular toxicity of new targeted (non-anthracycline) cancer therapies
[9]	Curigliano	2012	Definitions/management	ESMO oncology guidelines on cardiac monitoring, referral, and therapy
[10]	Christenson	2015	Early detection Biomarkers	Overview of circulating biomarkers in predicting chemotherapy-induced cardiac toxicity
[11]	Thavendiranathan	2014	Early detection Echocardiography	Echocardiographic myocardial deformation in the early detection of cardiotoxicity
[12]	Thavendiranathan	2013	Early detection CMR	The role of cardiac magnetic resonance in the detection of cardiotoxicity
[13]	Plana	2014	Imaging	ESC position paper on non-invasive imaging modalities in cardio-oncology
[14]	Herrmann	2014	Risk stratification and management	Practical aspects regarding cardio-oncology care, including an outline of a risk assessment tool
[15]	Zamorano	2016	Risk stratification and management	ESC position paper on cancer treatments and cardiovascular toxicity
[16]	Lancellotti	2013	Radiotherapy	Consensus paper on imaging and management of cardiovascular complications of radiotherapy
[5]	Naaktgeboren	2017	Long-term outcome	Overview on long-term outcome after anticancer treatment (chemo- and radiotherapy)
[17]	Dalen	2011	Prevention	Cochrane review on cardioprotective interventions for cancer patients receiving anthracyclines
[18]	Kalam	2013	Prevention	Systematic review on cardioprotective therapy for prevention of cardiotoxicity with chemotherapy
[19]	Johnson	2017	Training	Paper exploring training programs for medical specialists in cardio-oncology

## Scope of the problem

The incidence of CTRCD is determined by multiple factors, of which the most important involve the administered chemotherapeutic agent(s) and, in the case of anthracyclines, the cumulative dose. Additionally, specific patient characteristics have been shown to be associated with a higher risk of CTRCD. Anthracyclines and trastuzumab (Herceptin) are among the most widely prescribed agents associated with serious cardiotoxicity.

Anthracyclines (e. g. doxorubicin) are a cornerstone in the treatment of numerous haematological and solid malignancies. In a large meta-analysis pooling data from 18 studies involving a total of almost 50,000 patients undergoing treatment with anthracyclines, the incidence of clinically overt and subclinical cardiotoxicity was reported in 6.3% (3.2–9.3%) and 17.9% (11.6–24.2%) of patients respectively [20]. End-stage heart failure was observed in 2–4% of patients and carries a strikingly poor prognosis, with a 2-year mortality rate of up to 60% [21, 22].

Several attempts have been undertaken to reduce the incidence of anthracycline-induced cardiotoxicity. A dose-dependent relationship with heart failure led to restrictions in the administered cumulative dose. Other initiatives have involved the generation of numerous anthracycline analogues (e. g. epirubicin), concomitant administration of cardioprotective drugs (e. g. dexrazoxane), liposomal drug formulations, the application of prolonged infusion regimens to reduce peak plasma dose, and consecutive administration of other cardiotoxic drugs (i. e. trastuzumab), since simultaneous administration dramatically increases CTRCD incidence [18, 23–28]. Nevertheless, due to fear of impaired antitumour efficacy, the implementation of several of the above-mentioned preventive actions has been limited in clinical practice. Hence, anthracycline-related cardiotoxicity still remains a significant clinical problem [29].

Trastuzumab is administered in breast cancer patients with human epidermal growth factor receptor 2 (HER2) positive tumours [30]. A meta-analysis found an overall incidence of a left ventricular ejection fraction (LVEF) decline in 11.2% of patients (RR 1.83, 90% CI 1.36–2.47) [31]. Importantly, the prognosis of trastuzumab-induced cardiotoxicity is generally more favourable when compared to anthracycline-induced cardiotoxicity, with a recovery of LVEF after timely cessation of trastuzumab administration in a majority of patients [32].

## Definition

Multiple definitions of CTRCD have been proposed in the literature to date, although a consensus is currently still lacking [33]. The most widely adapted definition is a decrease in LVEF of more than 10 percentage points to a value below the lower limit of normal, irrespective of symptoms. The American Society of Echocardiography and the European Association of Cardiovascular Imaging (EACVI) define an LVEF of 53% on echocardiography as the lower limit of normal [13]. Subclinical CTRCD is defined as a global longitudinal strain (GLS) with >15% relative reduction from baseline with preservation of LVEF [13]. Unfortunately, this definition does not cover other signs of cardiotoxicity, such as the detection of cardiac troponin release.

## Pathophysiology

The pathophysiological mechanisms leading to CTRCD are complex, incompletely elucidated, and differ among chemotherapeutic agents [7, 8]. Traditionally, for agents that have a direct effect on cardiomyocytes, two types of cardiotoxicity have been proposed [24].

Type I cardiotoxicity is characterised by irreversible damage and related to the cumulative administered dose. Anthracyclines are most well-known for their association with type I cardiotoxicity. Mechanisms believed to play a role in this type of cardiotoxicity are multifactorial and involve (1) the generation of excess reactive oxygen species, (2) accumulation of toxic anthracycline metabolites that interfere with calcium handling and thereby disrupt sarcomere structure and function, (3) interaction with transcription factor topoisomerase-2β, and (4) mitochondrial dysfunction [6, 34, 35].

Type II cardiac damage is believed to cause temporary, reversible dysfunction in a dose-independent manner [32]. Trastuzumab is a classical type II agent, which binds to the HER2 receptor and thereby inhibits downstream associated signalling cascades. It is conceivable that the inhibition of these pathways plays a central role in trastuzumab-associated cardiotoxicity, but the exact mechanism still remains to be discovered [36].

Although the subdivision into type I and type II cardiotoxicity is arbitrary, as persistent LV dysfunction has also been observed in patients treated solely with type II agents, this subdivision is nevertheless still widely applied [37].

## Risk stratification

Cardiovascular management of patients receiving cardiotoxic treatment is based on the timely recognition of those at high risk of developing CTRCD. Accurate risk stratification is crucial to enable an effective pre-selection of patients that should be referred to a cardio-oncological team prior to, or in an early phase of, anticancer treatment.

### Treatment-related risk factors

Not all chemotherapeutic agents are (equally) cardiotoxic, as shown in Supplementary Table 1. In this table, we subdivided a subset of the most commonly used chemotherapeutic agents into four categories, based on the incidence of LV dysfunction reported in the literature (group 1: <1%, group 2: 1–5%, group 3: 5–10% and group 4: >10%). It should be noted that the applied definition of LV dysfunction varies between the studies, and that the incidence is an approximation of risk.

One of the most important risk factors for CTRCD in agents that cause type I cardiotoxicity is the administered dose. In patients that require more intensive regimens or have a history of previous malignancy for which they were treated with type I agents, it is important to take the cumulative dose into consideration. However, some patients develop CTRCD even with doses far below the maximum cumulative dose [23]. Therefore, a tolerated and ‘safe’ dose seems to be highly dependent on the presence of patient-related risk factors.

### Patient-related risk factors

Patient-related risk factors that have been identified thus far include female gender, black race, exposure to cardiotoxic drugs at a young or old age (<15 and >65 years), previous or concomitant chest radiation therapy, obesity and classical cardiovascular risk factors including hypertension and diabetes mellitus [15, 20, 38]. Having ≥3 of these risk factors has been associated with a 5–6 times higher risk of cardiotoxic side effects compared to patients without any risk factors [20]. Nonetheless, in the absence of all these known determinants, some patients still develop severe CTRCD, indicating that unknown factors contribute to individual susceptibility. It is conceivable that the individual genetic profile plays a considerable role in modulating individual risk [39].

### Risk prediction models

A few risk prediction models have been published in the literature thus far [14, 40, 41]. The Cardiotoxicity Risk Score (CRS) proposed by the Mayo Clinic takes both patient and treatment risk factors into account (Tab. 2; [14]). This model addresses the a priori risk of developing CTRCD. Patients with a CRS score of ≥4 could benefit from cardiological consultation during and after chemotherapeutic treatment, and those with high risk scores should be closely monitored during and after treatment. However, this risk model, as well as the other models, has not been validated in a prospective setting and its real clinical value remains to be determined. In the absence of validation, the European Society of Cardiology (ESC) therefore currently does not advise the use of a particular model in the position paper on cancer treatments and cardiovascular toxicity released in 2016 [15]. Instead, the committee stresses the importance of clinical judgement in individual risk assessment, which includes clinical history, physical examination and evaluation of cardiac function pre-chemotherapy.

**Table 2:** Cardiotoxicity Risk Score (*CRS*)

*Medication-related risk* ^*a*^		*Examples* ^*b*^
High (risk score 4)		Anthracyclines; trastuzumab; cyclophosphamide; 5‑fluorouracil
Intermediate (risk score 2)		Pertuzumab; vinblastine; capecitabine; ponatinib
Low (risk score 1)		Bevacizumab; imatinib
Rare (risk score 0)		Carboplatin; fludarabine; paclitaxel; rituximab
*Patient-related risk factors (1 point per item)*		
– Cardiomyopathy or heart failure – Coronary artery disease or equivalent (including peripheral artery disease) – Hypertension – Diabetes mellitus – Prior or concurrent anthracyclines – Prior or concurrent chest irradiation – Age <15 years or >65 years – Female gender		
*Overall risk by CRS and intensity of monitoring*		
>6 5–6 3–4 1–2 0	Very high High Intermediate Low Very low	

^a^The highest medication-related risk score (e. g. 4, 2, 1 or 0) is used for calculation of the CRS

^b^See the supplementary table for each separate agent and/or regime. Adapted from: [14]

## Early detection of myocardial damage

### Circulating biomarkers

**Fig. 1:**
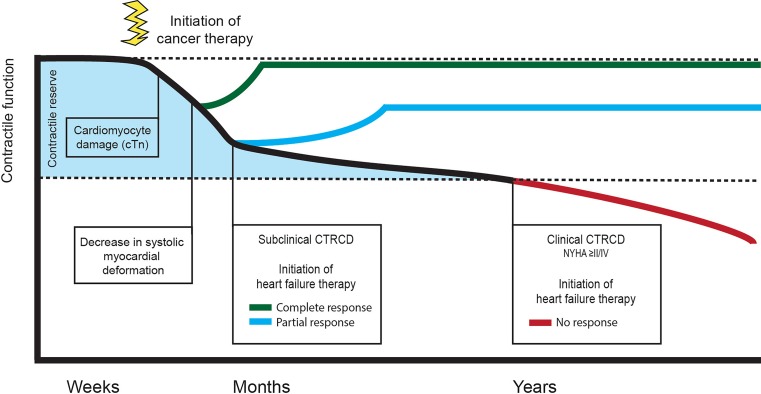
Time frame of detection and treatment of cardiotoxicity. Early initiation of heart failure treatment (*green*, *blue line*) leads to better outcomes regarding recovery of contractile function. Initiation of heart failure treatment at time when symptoms are present (*red line*) results in poor outcomes regarding recovery of cardiac function. *cTn* Cardiac troponin, *CTRCD* chemotherapy-related cardiac dysfunction, *NYHA* New York Heart Association classification

Due to the minimal invasiveness, limited costs, and low inter-observer variability, biomarkers constitute an appealing approach to aid in the early detection of subclinical cardiotoxicity (Fig. 1). Most studies have assessed the potential of classical cardiac biomarkers, i. e. cardiac troponin (cTn) and N‑terminal pro-B-type natriuretric peptide (NT-proBNP) [10]. Troponins seem to be the most promising candidates, both in patients treated with anthracyclines and various agents used for targeted-therapy (e. g. trastuzumab) [10, 42]. Nevertheless, repeated sampling is currently necessary to detect cTn elevations, as the optimal timing to reach maximal sensitivity has not yet been established [42].

### Multiple-gated acquisition scan

Since the 1970s the mainstay imaging modality for the screening and monitoring of cardiac function in oncology patients has been the multiple-gated acquisition scan [43]. Unfortunately, the only measurement that can be derived from these scans is the LVEF, which is less sensitive for early detection of CTRCD. Another important concern is the radiation exposure (~5–10 mSv/scan) in patients undergoing serial assessments. For example, the Dutch Guidelines for Breast Cancer recommend that patients receiving trastuzumab therapy should undergo cardiac evaluation to determine the LVEF before the start of treatment and subsequently once every 3 months during treatment [44]. The cumulative radiation exposure in patients treated with this agent for 1 year thereby equals ~25–50 mSv, which is comparable to 250–500 chest radiographs or 4–8 CT angiography procedures.

### (Strain) echocardiography

**Fig. 2:**
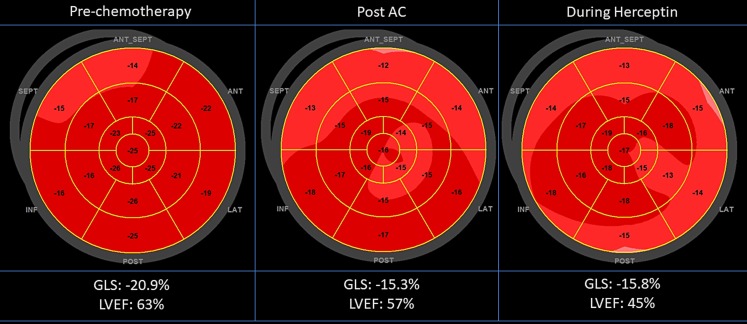
Echocardiographic deformation imaging. Longitudinal follow-up of a 51-year-old female with breast cancer with a high cardiovascular risk (Cardiotoxicity Risk Score 7: female, hypertension, concurrent anthracyclines, and high-risk agent trastuzumab). After the initial 4 × AC (adriamycin-cyclophosphamide) there was a significant decrease of >15% in global longitudinal strain (*GLS*) with preservation of left ventricular ejection fraction (*LVEF*). During the trastuzumab treatment there was a subsequent decrease in LVEF of >10%. After interruption of the trastuzumab treatment, the LVEF showed a complete recovery

Echocardiography is the most suitable imaging modality for the evaluation of patients in preparation for, during, and after cancer therapy, because of its wide availability, easy repeatability, versatility, lack of radiation exposure, and safety in patients with concomitant renal disease. Furthermore, echocardiography allows a comprehensive evaluation of most cardiac structures and multiple parameters besides the LVEF. It has been recommended as the first line screening tool to assess cardiac function in this specific patient population [13]. In particular, the measurement of LVEF by 3D echocardiography has been shown to be feasible and accurate with an error of <5% (compared to a 10% variation in biplane LVEF calculation) [45]. Echocardiographically derived GLS calculates the systolic deformation of the myocardium by a commercially available speckle tracking algorithm (Fig. 2; [46]). This parameter reflects contractile function and is well validated in healthy subjects and in a variety of myocardial disease states. The GLS has been shown to be the single best parameter to predict CTRCD, as a decrease of this parameter is often seen before a relevant reduction of LVEF is observed [11].

### Cardiac magnetic resonance

**Fig. 3:**
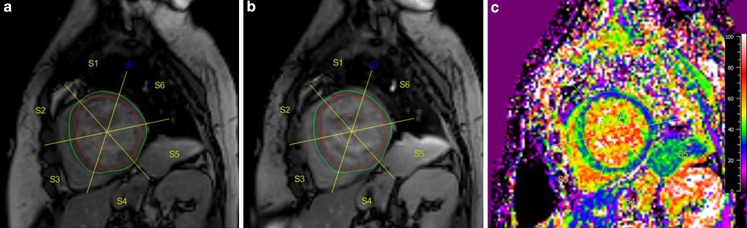
**a–c** Cardiac magnetic resonance imaging. Cardiac magnetic resonance imaging with T1 mapping in a female breast cancer survivor, treated with anthracyclines. Extracellular volume fraction (ECV) is a non-invasive measurement of diffuse myocardial fibrosis and can be calculated from the haematocrit; pre-contrast (**a**), post-contrast (**b**) T1 maps. In this patient, the ECV map (**c**) reveals diffuse elevated ECV values up to 42% (normal is <28%), in particular in the septal segments, reflecting widespread myocardial fibrosis after anthracycline exposure

Cardiac magnetic resonance (CMR) is considered the reference standard for the assessment of ventricular function [47]. Due to its superiority in myocardial tissue characterisation, CMR is suitable to detect early tissue damage following chemotherapy. Early changes in tissue composition include myocardial oedema with an increase in LV mass, inflammation, and decrease in myocardial strain [12]. Within months after initiation of therapy LV end-systolic volume increases, and with T1 mapping techniques diffuse interstitial fibrosis, a hallmark of anthracycline-induced cardiotoxicity, can be detected and quantified (Fig. 3a–c). The absence of focal fibrotic lesions results in a lack of late gadolinium enhancement, although contrast-enhanced CMR can be used to exclude other causes of myocardial dysfunction in these patients, such as myocardial infarction. Although unique insights can be obtained with MRI, due to costs and availability CMR is presently not suitable as the first-line imaging technique for regular follow-up imaging.

## Therapeutic interventions

With the exclusion of cancer patients in all high-impact heart failure intervention randomised controlled trials regarding the efficacy of, for example, ACE inhibitors and beta-blockers, the response rate of patients with CTRCD to conventional heart failure therapy has not been thoroughly investigated and evidence-based decision-making on optimal treatment is lacking [48].

**Fig. 4:**
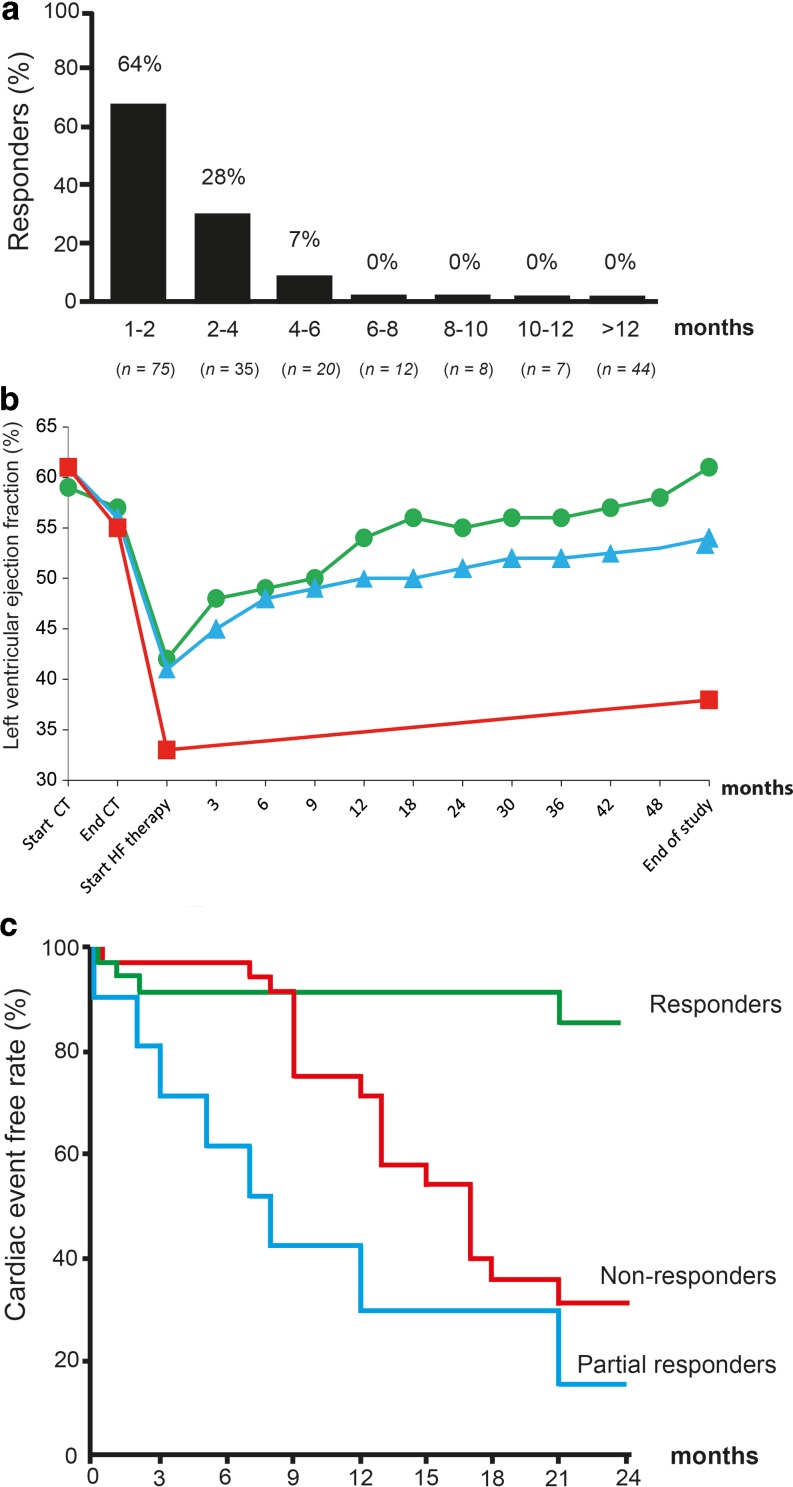
**a**–**c** Response and outcome to heart failure (*HF*) treatment in patients with chemotherapy-related cardiac dysfunction. **a** Percentage of (partial) responders according to the time elapsed from diagnosing left ventricular dysfunction and start of HF therapy. **b** Left ventricular ejection fraction in patients with cardiotoxicity and with no (*square/red*), partial (*triangle/blue*) or full (*dot/green*) recovery following heart failure therapy. **c** Cumulative cardiac event rate during follow-up. Reprinted from: [3, 49]. *CT* Chemotherapy

In a single-centre study by Cardinale et al. (*n* = 215), treatment response in patients with a decline of LVEF to ≤45% was highly dependent on the timing of treatment initiation [49]. The response rate was the highest (64% responders) among patients with CTRCD that received heart failure treatment <2 months after detection of LV impairment and decreased to only 7% after 4–6 months. Remarkably, no response was observed in patients with CTRCD that received treatment ≥6 months after the last chemotherapeutic cycle (Fig. 4a). Hence, detecting the development of CRTCD as soon as possible guides the optimal timing of treatment initiation, since this seems to be particularly crucial for treatment response. However, in a follow-up study by the same group, a large proportion of patients did not show recovery of cardiac function despite early initiation of conventional heart failure treatment. Only 11% showed full recovery to a mean LVEF of 61%. Cardiac function was partially restored in 71% of the patients, to a mean LVEF of 54%. Notably, 18% of patients did not respond to treatment, with a mean LVEF at the end of the study of 38% (Fig. 4b; [3]). Cardiac outcome for partial and non-responders is significantly worse, including heart failure requiring hospitalisation and cardiac-related death (Fig. 4c; [49]).

Currently, the only practice guidelines on the management of cardiovascular toxicity induced by anticancer treatment have been released by the European Society of Medical Oncology (ESMO) [9]. Guidelines from the ESC and the American Heart Association are still lacking, albeit the ESC recently released the first position paper on cancer treatments and cardiovascular toxicity [15].

The evidence for the use of cardioprotective agents to counteract LVEF decline in patients treated with anthracyclines is marginal, with the exception of dexrazoxane [17, 50]. Cardioprotective agents to prevent trastuzumab-related cardiotoxicity are largely unexplored. The results of the first randomised controlled trials investigating the cardioprotective effect of candesartan and carvedilol were recently published, showing no protection against LVEF decline [51].

## Roadmap towards outpatient management

**Fig. 5:**
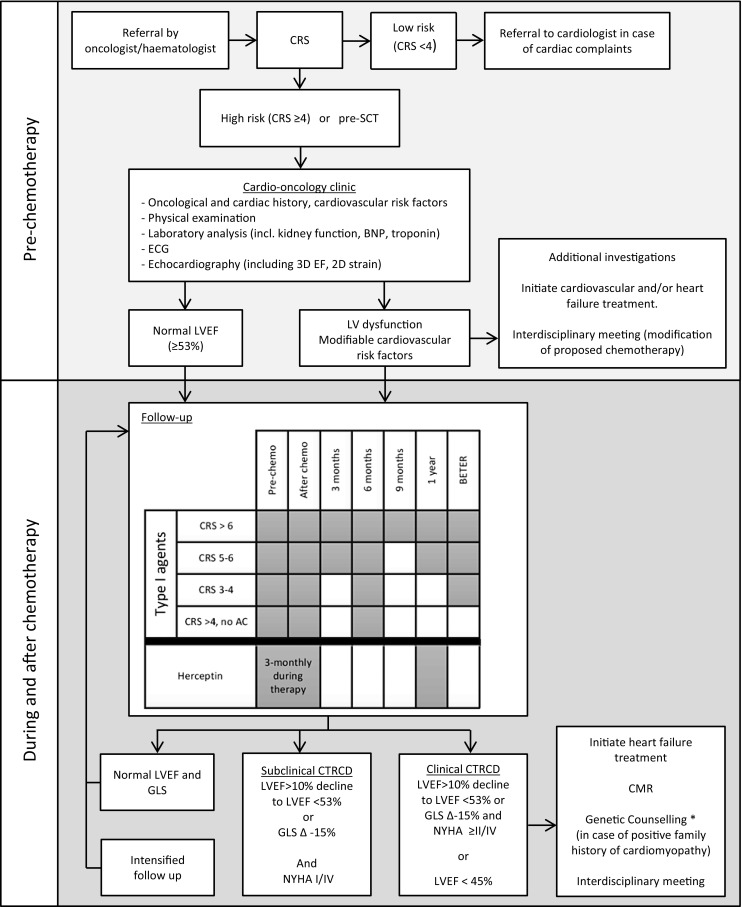
Cardio-oncology care at the University Medical Centre Utrecht, The Netherlands. *AC* Anthracyclines; *BNP* brain natriuretic peptide; *CMR* cardiac magnetic resonance; *CRS* Cardiotoxicity Risk Score; *CTRCD* chemotherapy-related cardiac dysfunction; *GLS* global longitudinal strain; *LVEF* left ventricular ejection fraction; *SCT* stem-cell transplantation. *To be considered, depending on local policy

Collaboration between the Departments of Cardiology, Radiology, Haematology and Oncology resulted in a specialised cardio-oncology healthcare pathway, which was launched at the University Medical Centre Utrecht, the Netherlands early in 2015. The aim of this initiative is to improve cardiac outcome in oncology patients by (1) identifying patients at high risk of developing CTRCD before chemotherapeutic treatment is initiated, (2) screen and monitor high-risk patients to enable early detection of (subclinical) cardiac dysfunction which (3) facilitates early treatment initiation in order to improve overall cardiovascular outcome. Patients are monitored up to 1 year after the end of chemotherapy, as a majority of the patients described in the literature develop CTRCD within this time frame [3]. It should be noted that long-term follow-up data in these patients are scarce. Our in-house protocol is delineated below (Fig. 5).

### Registration procedure

Patients are referred by the oncologist/haematologist based on in-hospital protocols on cardio-oncology referral. Main indications for referral are planned treatment with cardiotoxic agents, cardiac evaluation before (autologous or allogenic) stem-cell transplantation (SCT), or patients presenting with complaints suggestive of underlying cardiac disease (e. g. heart failure, ischaemia, or arrhythmias). The baseline risk is determined based on the Cardiotoxicity Risk Score (CRS) (Tab. 2). This risk score has been slightly adjusted from its initial publication [14] regarding the risk attributed to the different chemotherapeutic agents (see Supplementary table). Albeit this risk model has not been prospectively validated, we have chosen to incorporate it in our in-house protocol to ensure objective and uniform risk assessment and limit inter-physician variability. All patients pre-SCT as well as high-risk patients (CRS ≥ 4) that will have to undergo treatment with cardiotoxic agents are seen at our cardio-oncology outpatient clinic. Childhood cancer survivors and ex-Hodgkin patients are referred to the LATER and BETER [Dutch for ‘better’] outpatient clinic respectively [52].

### Initial cardiac evaluation

Preferentially, the first cardiac evaluation is performed before chemotherapeutic treatment is initiated. This first assessment involves exploration of the cardiac and oncological history (including a detailed assessment of known cardiovascular risk factors as well as past and planned chemotherapeutic regimens), physical examination, laboratory analysis (including kidney function, NT-proBNP and cTn), and an ECG. Furthermore, a complete echocardiographic evaluation is performed to determine cardiac dimensions, valvular function, LVEF (preferably 3D), GLS, diastolic function, right ventricular (RV) function and RV systolic pressure. Reference values can be obtained from the current EACVI guidelines [13]. In short, an LVEF of >53% on 2D/3D echocardiography and a GLS of −19.7% (−20.4% to −18.9%) are considered normal.

### Individual follow-up

The interval of follow-up for patients who are treated with agents associated with type I cardiotoxicity is determined by the baseline CRS (Fig. 5). The follow-up duration at the cardio-oncology clinic is typically up to 1 year after the last cycle of chemotherapy. After this period, further cardiac assessment takes place at the BETER outpatient clinic if the patient is found to be eligible [52]. Patients treated with trastuzumab are seen pre-chemotherapy, once every 3 months during treatment, and 1 year after the end of treatment. In asymptomatic patients that develop subclinical CTRCD, the monitoring interval is intensified as these subjects are at high risk for developing heart failure.

### Additional examinations

CMR is recommended in patients with clinical CTRCD and can be considered in patients with subclinical CTRCD. Measurements include LVEF, RVEF, delayed enhancement imaging, T_1rho_-mapping, and determination of GLS. Ischaemia detection using adenosine stress imaging is performed if there is a history of coronary artery disease, suspected ischaemia, or ≥2 cardiovascular risk factor(s) (smoking, diabetes, hypertension, hypercholesterolaemia and a positive family history) in men >40 years and post-menopausal women. Patients with severe CTRCD (LVEF < 45%) or a positive family history for cardiomyopathies are offered a referral for genetic counselling as part of our local policy, as genetic variants in cardiomyopathy-associated genes may have predisposed these patients to CTRCD [53, 54].

### Management of patients with CTRCD

Multidisciplinary meetings attended by the cardio-oncologist and oncologist/haematologist take place to discuss cases with (subclinical) CTRCD. At these meetings, the effect of modification(s) to the chemotherapeutic regimen to decrease cardiovascular toxicity is weighed against the consequences of these alterations for the oncological prognosis. Furthermore, in patients that have LV dysfunction prior to the initiation of cancer treatment, the optimal treatment regimen is chosen through shared decision-making.

#### Type I cardiac dysfunction

In patients with clinical CTRCD, treatment with ACE inhibition (preferentially enalapril) and a beta-blocker (preferentially carvedilol) is indicated. In patients with contraindications for ACE inhibition, an angiotensin-receptor blocker can be considered. Patients that show signs/symptoms of congestion receive loop diuretics. These recommendations are based on two single-centre studies [3, 49]. The value of other agents or the optimal dose are currently unknown. At the moment, there is no evidence that treatment with other heart failure drugs (e. g. aldosterone antagonists) has any added value.

#### Type II cardiac dysfunction

Upon development of type II cardiac dysfunction, the causal chemotherapeutic agent(s) should be discontinued immediately. After 3–4 weeks, the LVEF and GLS are re-assessed. If LV function has recovered, a re-challenge with the same chemotherapeutic agent(s) can be attempted under strict cardiological monitoring. Even though there are currently no evidence-based recommendations for heart failure medication in patients with this type of cardiotoxicity, treatment according to the ESC and ESMO guidelines should be considered if there is a persistent decline in LVEF and signs/symptoms of decompensation [9, 48].

#### Subclinical cardiac dysfunction

At this point in time, there is no evidence that initiation of heart failure medication in patients with subclinical cardiac dysfunction improves outcome. To prevent unnecessary treatment, we have decided to wait with initiation of heart failure treatment until the patient develops signs of clinical CTRCD, during the intensified follow-up.

## Future perspectives

Despite the advantages in our understanding of this specific heart-failure entity with regard to the underlying pathophysiological mechanisms, improving diagnostic accuracy, and implementation of specific therapeutic interventions, there are still several unresolved issues and challenges within the field of cardio-oncology. To detect opportunities for improvement at this moment in time, the routinely provided cardiovascular care in oncology patients prior to the cardio-oncology era has to be investigated. This includes the frequency at which baseline cardiac function is assessed, the incidence of cardiovascular complications, referral patterns, treatment initiation- and response. We acknowledge that the (adjusted) CRS is probably insufficient to accurately identify high-risk patients. Development of sophisticated algorithms, which can be applied in the clinical field, will be an important focus of future trials and registries in order to optimise resources and pursue a cost-effective health care system. Risk stratification models need prospective validation and further improvement by the identification of additional (genetic) risk factors. Furthermore, personalised chemotherapeutic regimens, with their increasing complexity, go hand in hand with the need to establish interactions between agents and the combined effect on the cardiovascular system. Early detection of subclinical cardiac damage and dysfunction seems essential to optimise the treatment response rate; therefore, suitable biomarkers as well as the timing of biomarker sampling and echocardiographic monitoring need to be investigated. Ongoing therapeutic trials (Tab. 3) will shed more light on the potential of conventional heart failure treatment in this population as well as the optimal timing of treatment initiation. The establishing of specialised cardio-oncology units across the Netherlands will speed the development of this field, optimising the cost-benefit ratio of chemotherapeutic treatment with the potential to improve both oncological and cardiac outcome [19, 55]. Furthermore, we will launch the ONCOR prospective multicentre registry in the near future, in which we aim to collect information on patients visiting cardio-oncology units across the Netherlands. Information from this registry will enable further national and international studies to improve the prognosis of this patient population.

**Table 3:** Ongoing clinical trials on the treatment of cardiotoxicity

Location	NCT number	Title	Intervention	Start date	
*USA*	NCT02943590	STOP-CA (Statins TO Prevent the Cardiotoxicity from Anthracyclines)	Atorvastatin or placebo	January 2017	Recruiting
*USA*	NCT02674204	STOP Heart Disease in Breast Cancer Survivors Trial	Atorvastatin or placebo	May 2016	Recruiting
*USA*	NCT02096588	Detection and Prevention of Anthracycline-Related Cardiac Toxicity with Concurrent Simvastatin	Simvastatin or placebo	May 2014	Active, not recruiting
*Canada*	NCT03186404	Statins for the Primary Prevention of Heart Failure in Patients Receiving Anthracycline Pilot Study	Atorvastatin or placebo	July 2017	Not yet recruiting
*UK*	NCT03265574	PROACT: Can We Prevent Chemotherapy-Related Heart Damage in Patients with Breast Cancer?	Enalapril or placebo	September 2017	Not yet recruiting
*Italy*	NCT01968200	Prevention of Anthracycline-Induced Cardiotoxicity	Enalapril	December 2012	Active, not recruiting
*USA*	NCT02177175	Carvedilol for the Prevention of Anthracycline/Anti-HER2 Therapy Associated Cardiotoxicity among Women with HER2-Positive Breast Cancer Using Myocardial Strain Imaging for Early Risk Stratification	Carvedilol or placebo	June 2014	Active, not recruiting
*Brazil*	NCT01724450	Carvedilol Effect in Preventing Chemotherapy-Induced Cardiotoxicity	Carvedilol or placebo	June 2012	Recruiting
*USA*	NCT02717507	Carvedilol in Preventing Heart Failure in Childhood Cancer Survivors	Carvedilol or placebo	April 2016	Recruiting
*USA*	NCT01347970	Pharmacologic Reversal of Ventricular Remodeling in Childhood Cancer Survivors at Risk for Congestive Heart Failure (PREVENT-CHF): A Phase IIB Randomized Placebo-Controlled Trial	Carvedilol or placebo	May 2012	Active, not recruiting
*Italy*	NCT02236806	Cardiotoxicity Prevention in Breast Cancer Patients Treated with Anthracyclines and/or Trastuzumab	Bisoprolol or ramipril or placebo	July 2015	Recruiting
*Canada*	NCT01016886	Multidisciplinary Approach to Novel Therapies in Cardiology Oncology Research	Perindopril or bisoprolol or placebo	September 2010	Active, not recruiting
*USA*	NCT01009918	Lisinopril or Coreg CR® in Reducing Side Effects in Women with Breast Cancer Receiving Trastuzumab	Carvedilol or lisinopril or placebo	March 2010	Active, not recruiting

## Electronic Supplementary Material: Cardiac toxicity for seperate anti-cancer agents

In the supplementary file we have provided the absolute risk (and clinical manifestations) for the most common 75 anti-cancer agents (sheet 1) with the corresponding references (sheet 2). The classification according to our modified CRS is provided (0 points = green, 1 point = yellow, 2 points = orange, 4 points = red). Furthermore, the abbreviations of the most commonly encountered 50 combinations of these agents are provided (sheet 3).

## References

[1] de Moor JS, Mariotto AB, Parry C (2013). Cancer survivors in the United States: prevalence across the survivorship trajectory and implications for care. Cancer Epidemiol Biomarkers Prev.

[2] Verdecchia A, Francisci S, Brenner H (2007). Recent cancer survival in Europe: a 2000–02 period analysis of EUROCARE-4 data. Lancet Oncol.

[3] Cardinale D, Colombo A, Bacchiani G (2015). Early detection of anthracycline cardiotoxicity and improvement with heart failure therapy. Circulation.

[4] Cardinale D (1996). A new frontier: cardio-oncology. Cardiologia.

[5] Naaktgeboren WR, Linschoten M (2017). de Graeff, et al. Cardiovascular health in adult cancer survivors. Maturitas.

[6] Rochette L, Guenancia C, Gudjoncik A (2015). Anthracyclines/trastuzumab: new aspects of cardiotoxicity and molecular mechanisms. Trends Pharmacol Sci.

[7] Lenneman CG, Cardio-oncology SDB (2016). an update on cardiotoxicity of cancer-related treatment. Circ Res.

[8] Moslehi JJ (2016). Cardiovascular toxic effects of targeted cancer therapies. N Engl J Med.

[9] Curigliano G, Cardinale D, Suter T (2012). Cardiovascular toxicity induced by chemotherapy, targeted agents and radiotherapy: ESMO Clinical Practice Guidelines. Ann Oncol.

[10] Christenson ES, James T, Agrawal V, Park BH (2015). Use of biomarkers for the assessment of chemotherapy-induced cardiac toxicity. Clin Biochem.

[11] Thavendiranathan P, Poulin F, Lim KD (2014). Use of myocardial strain imaging by echocardiography for the early detection of cardiotoxicity in patients during and after cancer chemotherapy: a systematic review. J Am Coll Cardiol.

[12] Thavendiranathan P, Wintersperger BJ, Flamm SD (2013). Cardiac MRI in the assessment of cardiac injury and toxicity form cancer chemotherapy: a systematic review. Circ Cardiovasc Imaging.

[13] Plana JC, Galderisis M, Barac A (2014). Expert consensus for multimodality imaging evaluation of adult patients during and after cancer therapy: a report from the American Society of Echocardiography and the European Association of Cardiovascular Imaging. J Am Soc Echocardiogr.

[14] Herrmann J, Lerman A, Sandhu NP, Villarraga HR, Mulvagh SL, Kohli M (2014). Evaluation and management of patients with heart disease and cancer: cardio-oncology. Mayo Clin Proc.

[15] Zamorano JL, Lancellotti P, Rodriguez Muñoz D (2016). 2016 ESC Position Paper on cancer treatments and cardiovascular toxicity developed under the auspices of the ESC Committee for Practice Guidelines: The Task Force for cancer treatments and cardiovascular toxicity of the European Society of Cardiology (ESC). Eur Heart J.

[16] Lancellotti P, Nkomo VT, Badano LP (2013). Expert consensus for multi-modality imaging evaluation of cardiovascular complications of radiotherapy in adults: a report from the European Association of Cardiovascular Imaging and the American Society of Echocardiography. J Am Soc Echocardiogr.

[17] van Dalen EC, Caron HN, Dickinson HO, Kremer LC (2011). Cardioprotective interventions for cancer patients receiving anthracyclines. Cochrane Database Syst Rev.

[18] Kalam K, Marwick TH (2013). Role of cardioprotective therapy for prevention of cardiotoxicity with chemotherapy: a systematic review and meta-analysis. Eur J Cancer.

[19] Johnson MN, Steingart R, Carver J (2017). How to develop a cardio-oncology fellowship. Heart Fail Clin.

[20] Lotrionte M, Biondi-Zoccai G, Abbate A (2013). Review and meta-analysis of incidence and clinical predictors of anthracycline cardiotoxicity. Am J Cardiol.

[21] Yeh ETH (2004). Cardiovascular complications of cancer therapy: diagnosis, pathogenesis, and management. Circulation.

[22] Felker GM, Thompson RE, Hare JM (2000). Underlying causes and long-term survival in patients with initially unexplained cardiomyopathy. N Engl J Med.

[23] Von Hoff DD, Layard MW, Basa P (1979). Risk factors for doxorubicin-induced congestive heart failure. Ann Intern Med.

[24] Ewer MS, Ewer SM (2015). Cardiotoxicity of anticancer treatments. Nat Rev Cardiol.

[25] Minotti G, Menna P, Salvatorelli E, Cairo G, Anthracyclines GL (2004). molecular advances and pharmacologic developments in antitumor activity and cardiotoxicity. Pharmacol Rev.

[26] Rafiyath SM, Rasul M, Lee B, Wei G, Lamba G, Liu D (2012). Comparison of safety and toxicity of liposomal doxorubicin vs. conventional anthracyclines: a meta-analysis. Exp Hematol Oncol.

[27] van Dalen EC, van der Pal HJ, Kremer LC (2016). Different dosage schedules for reducing cardiotoxicity in people with cancer receiving anthracycline chemotherapy. Cochrane Database Syst Rev.

[28] Slamon DJ, Leyland-Jones B, Shak S (2001). Use of chemotherapy plus a monoclonal antibody against HER2 for metastatic breast cancer that overexpresses HER2. N Engl J Med.

[29] Nohria A. Prevention of cardiomyopathy in patients with cancer. JACC online: http://www.acc.org/latest-in-cardiology/articles/2016/09/29/13/25/prevention-of-cardiomyopathy-in-patients-with-cancer Consulted on 14 March 2017.

[30] Senkus E, Kyriakides S, Ohno S (2015). Primary breast cancer: ESMO clinical practice guidelines for diagnosis, treatment and follow-up. Ann Oncol.

[31] Mantarro S, Rossi M, Bonifazi M (2016). Risk of severe cardiotoxicity following treatment with trastuzumab: a meta-analysis of randomized and cohort studies of 29,000 women with breast cancer. Intern Emerg Med.

[32] Ewer MS, Vooletich MT, Durand JB (2005). Reversibility of trastuzumab-related cardiotoxicity: new insights based on clinical course and response to medical treatment. J Clin Oncol.

[33] Bloom MW, Hamo CE, Cardinale D (2016). Cancer therapy-related cardiac dysfunction and heart failure: Part 1: definitions, pathophysiology, risk factors, and imaging. Circ Heart Fail.

[34] Mushlin PS, Cusack BJ, Boucek RJ, Andrejuk T, Li X, Olson RD (1993). Time-related increases in cardiac concentrations of doxorubicinol could interact with doxorubicin to depress myocardial contractile function. Br J Pharmacol.

[35] Zhang S, Liu X, Bawa-Khalfe T (2012). Identification of the molecular basis of doxorubicin-induced cardiotoxicity. Nat Med.

[36] De Keulenaer GW, Doggen K, Lemmens K (2010). The vulnerability of the heart as a pluricellular paracrine organ: lessons from unexpected triggers of heart failure in targeted ErbB2 anticancer therapy. Circ Res.

[37] Telli ML, Hunt SA, Carlson RW, Guardino AE (2007). Trastuzumab-related cardiotoxicity: calling into question the concept of reversibility. J Clin Oncol.

[38] Krischer JP, Epstein S, Cuthbertson DD, Goorin AM, Epstein ML, Lipshultz SE (1997). Clinical cardiotoxicity following anthracycline treatment for childhood cancer: the Pediatric Oncology Group experience. J Clin Oncol.

[39] Linschoten M, Teske AJ, Cramer MJ, van der Wall E, Asselbergs FW (2018). Chemotherapy-related cardiac dysfunction—a systematic review of genetic variants modulating individual risk. Circ Genom Precis. Med.

[40] Dranitsaris G, Rayson D, Vincent M (2008). The development of a predictive model to estimate cardiotoxic risk for patients with metastatic breast cancer receiving anthracyclines. Breast Cancer Res Treat.

[41] Ezaz G, Long JB, Gross CP, Chen J (2014). Risk prediction model for heart failure and cardiomyopathy after adjuvant trastuzumab therapy for breast cancer. J Am Heart Assoc.

[42] Cardinale D, Biasillo G, Salvatici M, Sandri MT, Cipolla CM (2017). Using biomarkers to predict and to prevent cardiotoxicity of cancer therapy. Expert Rev Mol Diagn.

[43] Gottdiener JS, Mathisen DJ, Borer JS (1981). Doxorubicin cardiotoxicity: assessment of late left ventricular dysfunction by radionuclide cineangiography. Ann Intern Med.

[44] Breast Cancer Guideline version 2.0, NABON 2012: http://www.oncoline.nl/uploaded/docs/mammacarcinoom/Dutch%20Breast%20Cancer%20Guideline%202012.pdf Consulted on 30^th^ of March 2017.

[45] Thavendiranathan P, Grant AD, Negishi T (2013). Reproducibility of echocardiographic techniques for sequential assessment of left ventricular ejection fraction and volumes: application to patients undergoing cancer chemotherapy. J Am Coll Cardiol.

[46] Teske AJ, De Boeck BW, Melman PG, Sieswerda GT, Doevendans PA, Cramer MJ (2007). Echocardiographic quantification of myocardial function using tissue deformation imaging, a guide to image acquisition and analysis using tissue Doppler and speckle tracking. Cardiovasc Ultrasound. Acta Neurochir (Wien).

[47] Grothues F, Smith GC, Moon JC (2002). Comparison of interstudy reproducibility of cardiovascular magnetic resonance with two-dimensional echocardiography in normal subjects and in patients with heart failure or left ventricular hypertrophy. Am J Cardiol.

[48] Ponikowski P, Voors AA, Anker SD (2016). 2016 ESC Guidelines for the diagnosis and treatment of acute and chronic heart failure: The Task Force for the diagnosis and treatment of acute and chronic heart failure of the European Society of Cardiology (ESC). Developed with the special contribution of the Heart Failure Association (HFA) of the ESC. Eur J Heart Fail.

[49] Cardinale D, Colombo A, Lamantia G (2010). Anthracycline-induced cardiomyopathy: clinical relevance and response to pharmacologic therapy. J Am Coll Cardiol.

[50] Avila MS, Ayub-Ferreira SM, de Barros Wanderley MR (2018). Carvedilol for prevention of chemotherapy-related cardiotoxicity: the CECCY trial. J Am Coll Cardiol.

[51] Boekhout AH, Gietema JA, Milojkovic Kerklaan B (2016). Angiotensin II-receptor inhibition with candesartan to prevent trastuzumab-related cardiotoxic effects in patients with early breast cancer: a randomized clinical trial. Jama Oncol.

[52] Dekker N, van ‘t Veer MB, Aleman BM (2015). The BETER survivorship care initiative for Hodgkin lymphoma; a tailored survivorship care for late effects of treatment. Ned Tijdschr Geneeskd.

[53] Wasielewski M, van Spaendonck-Zwarts KY, Westerink ND (2014). Potential genetic predisposition for anthracycline-associated cardiomyopathy in families with dilated cardiomyopathy. Open Heart.

[54] Linschoten M, Teske AJ, Baas AF (2017). Truncating titin (TTN) variants in chemotherapy-induced cardiomyopathy. J Card Fail.

[55] Shee K, Kono AT, D’Anna SP (2016). Maximizing the benefit-cost ratio of anthracyclines in metastatic breast cancer: case report of a patient with a complete response to high-dose doxorubicin. Case Rep Oncol.

